# Combining extracellular volume fraction imaging and T2 quantification by cardiovascular magnetic resonance in patients with clinically suspected myocarditis

**DOI:** 10.1186/1532-429X-15-S1-P98

**Published:** 2013-01-30

**Authors:** Ulf K Radunski, Gunnar Lund, Fadi Obeidat, Christian Stehning, Gerhard Adam, Stefan Blankenberg, Kai Muellerleile

**Affiliations:** 1 University Heart Center Hamburg, Hamburg, Germany; 2 Diagnostic and Interventional Radiology, University Medical Center Hamburg-Eppendorf, Hamburg, Germany; 3 Philips Research Hamburg, Hamburg, Germany

## Background

Assessing the extracellular volume fraction (ECV) by T1 mapping and T2 quantification are promising quantitative cardiovascular magnetic resonance (CMR) tools in acute myocardial injury. This study evaluated the diagnostic value of combining ECV imaging and T2 quantification in patients with clinically suspected myocarditis.

## Methods

**Figure 1:**
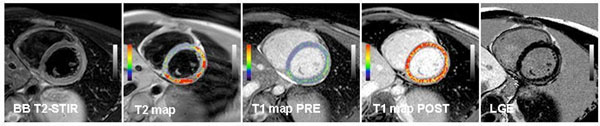


Short-axis T1 and T2 mapping were implemented into a standard CMR protocol at 1.5 Tesla in 36 patients with clinically suspected myocarditis. T1 quantification was performed for ECV calculation using the modified Look-Locker inversion-recovery (MOLLI) sequence before and 15 minutes after administration of 0.075 mmol/kg gadolinium BOPTA. T2 quantification was performed using a free-breathing, navigator-gated multi-echo sequence. T1 and T2 maps were calculated with a dedicated plug-in written for the OsiriX software. Relaxation rates (1/T1 = R1) were calculated for myocardium and blood pool. The difference in R1 between pre- and post contrast media was calculated as ΔR1. Myocardial ECV was then estimated using the formula: ECV = 1-hematocrit * (ΔR1_myocardium_/ΔR1_blood pool_). Figure 1 demonstrates an example for a T2 map and T1 maps pre-/post contrast media in comparison with standard black-blood T2 STIR and late gadolinium enhancement (LGE) images.

## Results

There was a moderate correlation between global ECV and global T2 values (r=0.51, p<0.01). Current clinical and CMR criteria identified 31 (86 %) patients with myocarditis and 5 (14 %) patients without myocarditis in this study population. Global ECV was significantly larger in patients with myocarditis (35 (32-39) %) compared to patients without myocarditis (26 (24-37) %; p<0.05). A non-significant difference in global T2 was found between patients with (61 (58-66) ms) and without myocarditis (55 (52-62) ms; p=0.08). Presence of a global ECV ≧ 32 % and/or a global T2 ≧ 58 ms provided positive and negative predictive values to identify patients with myocarditis of 94 and 60 %, respectively (p<0.05).

## Conclusions

Our findings indicate a potential incremental diagnostic value of combining ECV imaging and T2 quantification in patients with clinically suspected myocarditis.

## Funding

Orlovic Foundation

